# Toward One Health Institutionalization: harnessing stakeholder network as leverage to strengthen health security in Libya

**DOI:** 10.3389/fpubh.2025.1651901

**Published:** 2025-11-21

**Authors:** Asma Saidouni, Ramy Mohamed Ghazy, Abdulaziz Zorgani, Omar Elahmer, Diyaeddin Natuh, Ramadhan Mohamed Osman, Abdelaziz El Halafi, Ahmed Zouiten

**Affiliations:** 1 World Health Organization, Tripoli, Libya; 2 Tropical Health Department, High Institute of Public Health, Alexandria University, Alexandria, Egypt; 3 Family and Community Medicine Department, College of Medicine, King Khalid University, Abha, Saudi Arabia; 4 National Centre for Disease Control, Tripoli, Libya; 5 Department of Medical Microbiology, Faculty of Medicine, University of Tripoli, Tripoli, Libya; 6 Department of Dental Technology, Faculty of Medical Technology, University of Tripoli, Tripoli, Libya

**Keywords:** One Health, Libya, multisectoral coordination, collaboration, stakeholders mapping, health security, pandemic prevention, preparedness and response

## Abstract

**Background:**

The institutionalization of the One Health approach is critical for addressing complex health threats at the human-animal-environment interface. In Libya—a state affected by prolonged political conflict, the growing impact of climate change, and weak intersectoral coordination—such an approach is critical to address zoonotic diseases, antimicrobial resistance (AMR), and climate-related health threats. This study aimed to map and analyze stakeholder networks to inform the development of a national One Health governance framework in Libya.

**Methods:**

We employed a mixed-methods approach integrating participatory Net-Map stakeholder mapping, social network analysis (SNA), and SWOT analysis during a national consultation workshop (September 2024) with 42 multisectoral experts. SNA metrics (degree, betweenness, eigenvector centrality, modularity) were computed using R software to analyze a network of 11 core institutions and 102 directed ties across four interaction modalities: coordination, collaboration, capacity building, and advocacy.

**Results:**

The network was structurally cohesive (reciprocity = 0.857; average path length = 2.05) but functionally siloed into three clusters: (1) an Operational One Health Interface comprising the National Center for Disease Control (NCDC), National Center for Animal Health (NCAH), Environmental Sanitation Affairs (ESA), and Ministry of Environment (MoE); (2) an Agricultural and Livestock Governance Cluster including the Ministry of Agriculture (MoA), Food and Agriculture Organization of the United Nations (FAO), Ministry of Local Government (MoLG), and World Organization for Animal Health (WOAH); and (3) a Public Health and Regulatory Cluster consisting of the Ministry of Health (MoH), Food and Drug Control Center (FDCC), and World Health Organization (WHO). NCAH and NCDC emerged as central hubs, while MoA served as the key broker (betweenness centrality = 0.334). SWOT analysis identified strong technical expertise and centralized infrastructure as key strengths but highlighted fragmented coordination, limited funding, and political instability as major constraints.

**Conclusion:**

These evidence-based insights directly informed Libya’s first national One Health Memorandum of Understanding (MoU), establishing a formal governance framework signed by the MoH, MoA, MoLG, MoE, and FDCC, and endorsed by NCDC, the NCAH, and ESA. The study demonstrates that even in fragile contexts, network-informed stakeholder engagement can catalyze sustainable, multisectoral health governance—offering a replicable model for One Health institutionalization in similar settings as a catalyst for health security. It highlights practical lessons learned from the COVID-19 pandemic, underscoring how integrated governance across human, animal, and environmental health sectors can enhance prevention, preparedness, response, and resilience against future threats.

## 1 Introduction

The emergence and re-emergence of zoonotic diseases, driven by close human-animal contact, climate change, and modern agricultural practices, have propelled the One Health approach to global prominence (1). This crisis underscores the urgent need to move beyond sectoral silos and institutionalize One Health as a fundamental strategic direction for collective action aimed at mitigating future pandemic risks and strengthening health systems globally (2, 3). The One Health concept seeks to address complex health issues at the intersection of human, animal, and environmental health by integrating efforts from relevant sectors and disciplines and different organizational levels (4). This approach is crucial for addressing complex health issues and is increasingly recognized as key to ensuring collective efforts to mitigate pandemic risks and improve global health security.

The relevance of One Health is further underscored by its alignment with the United Nations Sustainable Development Goals (SDGs), which link health, water, climate, and ecosystem sustainability. To advance these goals, four major organizations—the World Health Organization (WHO), the World Organization for Animal Health (WOAH), the Food and Agriculture Organization of the United Nations (FAO), and the United Nations Environment Program (UNEP)—have formed the One Health Quadripartite alliance. They focus on six key areas: laboratory services, zoonotic disease control, neglected tropical diseases, antimicrobial resistance (AMR), food safety, and environmental health (5).

However, the implementation of One Health faces significant challenges, particularly in low-and middle-income countries (LMICs) with fragile governance (6). This is evident across diverse national contexts: Jordan has well-established ministerial infrastructures but struggles with inconsistent reporting, inadequate regulations, a limited surveillance system, and insufficient diagnostic capabilities for zoonotic diseases (7). Conversely, Uganda, a hotspot for epidemics, has formed a National One Health platform and developed strategic plans. However, it faces challenges related to weak coordination, inadequate government commitment, and a lack of advocacy and research (8). Similarly, Ethiopia has pioneered One Health through steering committees, prioritized zoonotic diseases, and joint outbreak investigations. Its main hurdles include poor sectoral integration in data sharing, a lack of institutionalization and sustainable government funding, and limited research (9).

Libya exemplifies these challenges; the nation’s extended period of political turmoil has resulted in significant challenges to centralized governance and diminished institutional capacity, creating a primary obstacle to the coordinated leadership and stable infrastructure required for One Health (10). Moreover, Libya is affected by extensive migration from sub-Saharan Africa, alongside unregulated animal movement and trade, which can introduce pathogens and disease vectors (11). Additionally, its position on the Mediterranean/Black Sea Flyway means migratory birds utilize Libyan wetlands as stopover sites, presenting another pathway for disease transmission (12). These factors increase the risk of the introduction of pathogens and disease vectors into the country, which in turn can lead to the emergence of zoonotic diseases. Within this vulnerable context, the threat of AMR is amplified by unrestricted access to antimicrobials, inefficient infection prevention and control, and in some areas, insufficient water, sanitation, and hygiene (WASH) programs (13–17). Beyond these structural and situational barriers, conceptual obstacles also hinder progress. These include deeply divided policymaking across human, animal, and environmental health sectors and a lack of consensus on the operational definition and scope of “One Health,” which leads to stakeholder uncertainty and obstructs the formulation of a cohesive national strategy (6). Therefore, this study aimed to support the effective institutionalization of the One Health approach in Libya. The specific objectives were to secure political commitment and enhance multi-sectoral collaboration. To achieve this, a stakeholder mapping exercise was conducted to identify key actors and assess their level of interest and influence regarding One Health. The insights from this analysis directly informed the development of a national Memorandum of Understanding (MoU) to formalize Libya’s One Health governance mechanism. In parallel, a SWOT (Strengths, Weaknesses, Opportunities, Threats) analysis was conducted to critically assess the internal and external factors affecting One Health institutionalization. Together, these initiatives provide a foundational strategy for operationalizing One Health in Libya, aligning stakeholders around a shared vision, and enabling context-specific planning for sustainable implementation.

## 2 Methodology

This cross-sectional study employed a mixed-methods approach to establish a foundational framework for One Health institutionalization in Libya, integrating participatory stakeholder mapping, social network analysis (SNA), and a SWOT analysis. The stakeholder mapping method tailored to One Health was developed within the operational framework of the Capacitating One Health in Eastern and Southern Africa (COHESA) project. The COHESA consortium—comprising the International Livestock Research Institute (ILRI), the French Agricultural Research Center for International Development (CIRAD), and the International Service for the Acquisition of Agri-biotech Applications (ISAAA AfriCenter)—provided overarching technical support and regional coordination. The adaptation and application of the Net-Map methodology to the Libyan context were specifically led by the WHO Libya country office, ensuring methodological rigor and alignment with broader One Health institutionalization efforts.

### 2.1 Workshop design and participant composition

A national One Health consultation workshop was convened in Tripoli, Libya, from September 3–5, 2024, to facilitate multisectoral collaboration. To ensure methodological validity, participants were selected through purposive sampling, prioritizing individuals with in-depth expertise, direct operational experience, and demonstrated engagement in human, animal, or environmental health domains. Additional criteria included availability, willingness to participate, and ability to articulate insights clearly consistent with established qualitative research standards. The workshop brought together 42 key stakeholders from governmental ministries, national technical agencies, and academic institutions. Participant distribution was as follows: National Center for Disease Control (NCDC, *n* = 9), National Center for Animal Health (NCAH, *n* = 7), Ministry of Health (MoH, *n* = 6), Ministry of Environment (MoE, *n* = 5), Food and Drug Control Center (FDCC, *n* = 5), Environmental Sanitation Affairs (ESA, *n* = 5), Ministry of Agriculture (MoA, *n* = 2), and academic institutions (*n* = 3). Participants represented diverse disciplines—including public health, epidemiology, veterinary medicine, laboratory sciences, food safety, environmental health, and climate change—ensuring multidisciplinary and multisectoral representation aligned with the One Health approach.

### 2.2 Goal definition and strategic objectives

The overarching goal of the initiative was to establish a formal and sustainable One Health governance framework in Libya. To operationalize this, a multisectoral task force was formed with the mandate to develop a national MoU. Before the workshop, the task force agreed on three evidence-based analytical objectives:

1. Identify key stakeholders capable of driving One Health institutionalization.
2. Conduct a situational review of national regulations related to zoonotic/vector-borne diseases, food safety, and AMR.

### 2.3 Stakeholder identification and influence-interest analysis

**Figure 1:**
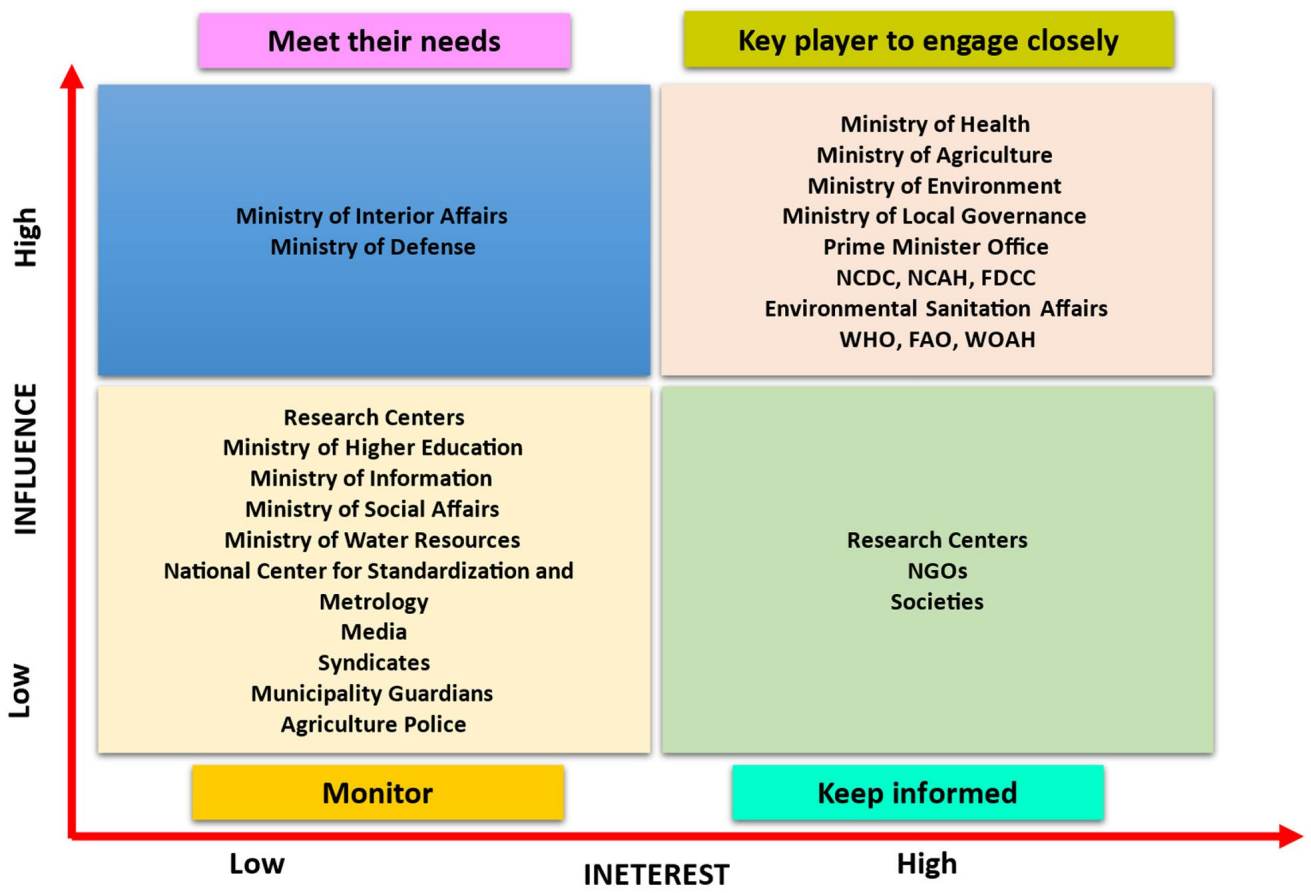
Stakeholder grid showing influence and interest matrix.

Using the Net-Map tool—a participatory social network analysis method developed by the International Food Policy Research Institute (IFPRI) (18), workshop participants systematically identified 27 key actors across four categories (Figure 1):

- Ministries (e.g., MoH, MoA, MoE, MOLG).
- Government agencies (e.g., NCDC, NCAH, FDCC, ESA).
- International partners (e.g., WHO, FAO, and WOAH).
- National associations and academic institutions.

These stakeholders were then plotted on an influence-interest matrix based on two dimensions:

- Influence: capacity to affect decisions through formal authority, expertise, or resource control.
- Interest: level of concern or vested stake in One Health outcomes.

This matrix enabled strategic stakeholder segmentation and guided tailored engagement strategies (e.g., “Manage Closely,” “Keep Satisfied”).

### 2.4 Defining inter-stakeholder linkages

Building on the influence-interest analysis, participants defined functional relationships among stakeholders identified as key actors for the One Health institutionalization in Libya through a structured plenary session. The Prime Minister’s Office was not included in this analysis due to its unique, overarching convening role. Actors were first categorized by role:

- Core institutional drivers: entities with formal authority and budgetary power (e.g., ministries).
- Implementation enablers: organizations providing technical or operational support (e.g., Civil Society Organizations, academia).

Four key linkage types were operationally defined and prioritized for institutionalization:

- Collaboration: formal partnerships, often codified by agreements.
- Coordination: joint planning and synchronized action.
- Capacity building: skill and resource development across sectors.
- Advocacy: efforts to secure political buy-in and raise awareness.

Participants mapped existing communication channels, interaction frequency, directionality, and resource flows, identifying both leverage points and critical gaps in the current network.

### 2.5 Visualization of linkages and influence mapping

Participants were divided into four thematic groups, each assigned to map one linkage type using color-coded directional ties. Arrows indicated direction of influence or support; bidirectional arrows denoted mutual engagement. “Influence towers” (constructed using Lego® bricks) visually represented each actor’s relative influence based on the number and strength of incoming ties. The resulting physical maps were digitized using network visualization software to produce dynamic diagrams for further analysis.

### 2.6 Social network analysis (SNA)

To complement the participatory mapping, a quantitative SNA was performed on a dataset of 11 core stakeholders (identified as having High Influence and High Interest) and 102 directed ties across the four linkage modalities. The analysis was executed using R software (version 4.3.1). The multi-edge weighted network was used, where edge weights represented the count of distinct interaction modalities (coordination, collaboration, capacity building, advocacy) between stakeholders.

Network construction, analysis, and metric computation were carried out using the igraph package. The following metrics were calculated to empirically validate the participatory findings and reveal nuanced stakeholder roles:

- Node-level metrics: Influence and activity (in-degree, out-degree, weighted degree); brokerage and structural autonomy (betweenness centrality, calculated using Brandes’ algorithm, and constraint); and integration and reach (closeness centrality, eigenvector centrality [power iteration method], and Node-level metrics included measures of influence and activity (in-degree, out-degree, weighted degree), brokerage and structural autonomy (betweenness centrality, computed using Brandes’ algorithm, and constraint), and integration and reach (closeness centrality, eigenvector centrality using the power iteration method, and PageRank).
- Network-level metrics: Structural properties including density, reciprocity, average path length, transitivity, and assortativity.

Network visualization was achieved using the ggraph package. The tidyverse suite was used for data wrangling, while scales, knitr, and kableExtra enhanced the clarity of data presentation and the generation of structured results tables.

#### 2.6.1 Operational definition

The following metrics were computed to quantify stakeholder roles and network structure:

- Degree centrality: Calculated using degree (), this metric measures the total number of direct connections (ties) a stakeholder (node) has with other stakeholders in the network. A high degree of centrality indicates that an institution is highly active in interactions, either initiating or receiving linkages across collaboration, coordination, capacity building, or advocacy. It reflects the breadth of engagement.
- Weighted degree (Strength): The weighted degree of a node is calculated by summing the weights (frequencies or intensities) of all ties associated with it, counting each tie once for each interaction type (for instance, if a stakeholder pair is connected through both coordination and capacity building, it contributes 2 to the weighted degree). This measure reflects both the intensity and diversity of connections. A large, weighted degree indicates substantial, varied involvement.
- Betweenness centrality: Computed using betweenness(), this metric measures the share of shortest paths between other node pairs that pass through a specific node. Nodes with high betweenness act as brokers or bridges, linking groups that would otherwise remain disconnected. Such stakeholders play a pivotal role in facilitating information flow and promoting cross-sectoral integration.
- Broker score: This metric quantifies the proportion of a node’s interactions that serve as bridges between different predefined subgroups or sectors within the network. It provides a direct measure of an actor’s role in facilitating cross-sectoral exchange and integration.
- Constraint: Calculated using constraint(), it measures the extent to which a node’s connections are concentrated to a single neighbor or a small group of interconnected neighbors. It quantifies the limitation of a node’s brokerage potential by its own network environment.
- Closeness centrality: The reciprocal of the average shortest path length from a node to all other nodes in the network. Stakeholders with high closeness centrality can quickly access or influence the entire network, positioning them effectively for timely coordination, rapid information dissemination, and swift response mobilization.
- Eigenvector centrality: Computed using eigen_centrality(), it is a measure of a node’s influence that accounts for the importance of its connections, giving higher weight to links with well-connected nodes than to those with less-connected ones. Stakeholders with high eigenvector centrality are tied to other influential actors, reflecting not just activity but strategic positioning within the network’s core of power, where influence flows through association.
- PageRank: Calculated using page_rank(), it is a variant of eigenvector centrality that calculates the likelihood of reaching a node through random walks across the network, incorporating a damping factor to reflect the network’s structure. PageRank identifies stakeholders with sustained structural importance, capturing both direct and indirect influence, and demonstrating enduring centrality even in complex network environments.
- Community Structure (Modularity-Based Clustering): The division of a network into subgroups (communities) characterized by denser connections within groups than between them, typically identified using the Louvain algorithm. These communities often mirror functional or sectoral alignments. Analyzing such clusters reveals natural pathways of collaboration as well as gaps between silos that may require intentional bridging.

### 2.7 SWOT analysis for strategic planning

A structured, multi-stage qualitative consensus process was employed to conduct the SWOT analysis. Participants were divided into four thematic working groups, each assigned to systematically identify factors for one of the four SWOT categories. The analysis was guided by a standardized framework of prompting questions aligned with the study’s objectives. For instance, groups considered questions such as: “What existing policies, skills, or infrastructure give Libya an advantage in One Health?” (Strengths); “What gaps in coordination, funding, or awareness hinder progress?” (Weaknesses); “What external support, partnerships, or global initiatives can be leveraged?” (Opportunities); and “What political, economic, or environmental pressures could threaten success?” (Threats). Following in-depth group discussions, the findings were presented in a plenary session where each factor was reviewed, debated, and validated through formal consensus voting to ensure only universally acknowledged items were retained. A pre-defined threshold of >70% participant agreement was required for a factor to be included in the final SWOT matrix. To strengthen internal validity and contextual relevance, the consolidated findings were cross-referenced with SNA results. This rigorous process ensured that the final SWOT matrix directly and reliably informed the strategic priorities embedded in the national One Health MoU.

## 3 Results

### 3.1 Influence–interest analysis

Actors were plotted on a stakeholder influence/interest matrix (Figure 1), which serves as a strategic visual tool for understanding their relative potential impact on and commitment to the One Health initiative. The matrix revealed distinct stakeholder segments, guiding targeted engagement strategies:

- High Influence, High Interest (“Manage Closely”): This pivotal group, including the MoH, MoA, MoE, MoLG, Prime Minister’s Office, NCDC, NCAH, FDCC, ESA, WHO, FAO, and WOAH, is essential for both policy formulation and execution, necessitating continuous and close collaboration.
- High Influence, Low Interest (“Keep Satisfied”): Entities such as the Ministry of Interior Affairs (MoIA) and Ministry of Defense (MoD) wield significant authority but have lower direct interest. Engagement should focus on meeting their specific needs to secure their support.
- Low Influence, High Interest (“Keep Informed”): Comprising research centers, the Ministry of Higher Education (MoHE), non-governmental organizations (NGOs), and professional or civil societies, these stakeholders are strong allies. Keeping them well-informed fosters advocacy and broad-based support.
- Low Influence, Low Interest (“Monitor”): Stakeholders like the Ministry of Information (MoI) and Ministry of Social Affairs (MoSA) require minimal effort but should be monitored for potential risks or emerging opportunities.

The resulting matrix served as a strategic framework to prioritize engagement, highlighting influential stakeholders whose buy-in was critical for driving the initiative forward and identifying entities requiring targeted communication. This approach enabled the systematic development of tailored strategies to secure broad-based, multi-sectoral commitment.

### 3.2 Social network structure and centrality metrics

**Figure 2:**
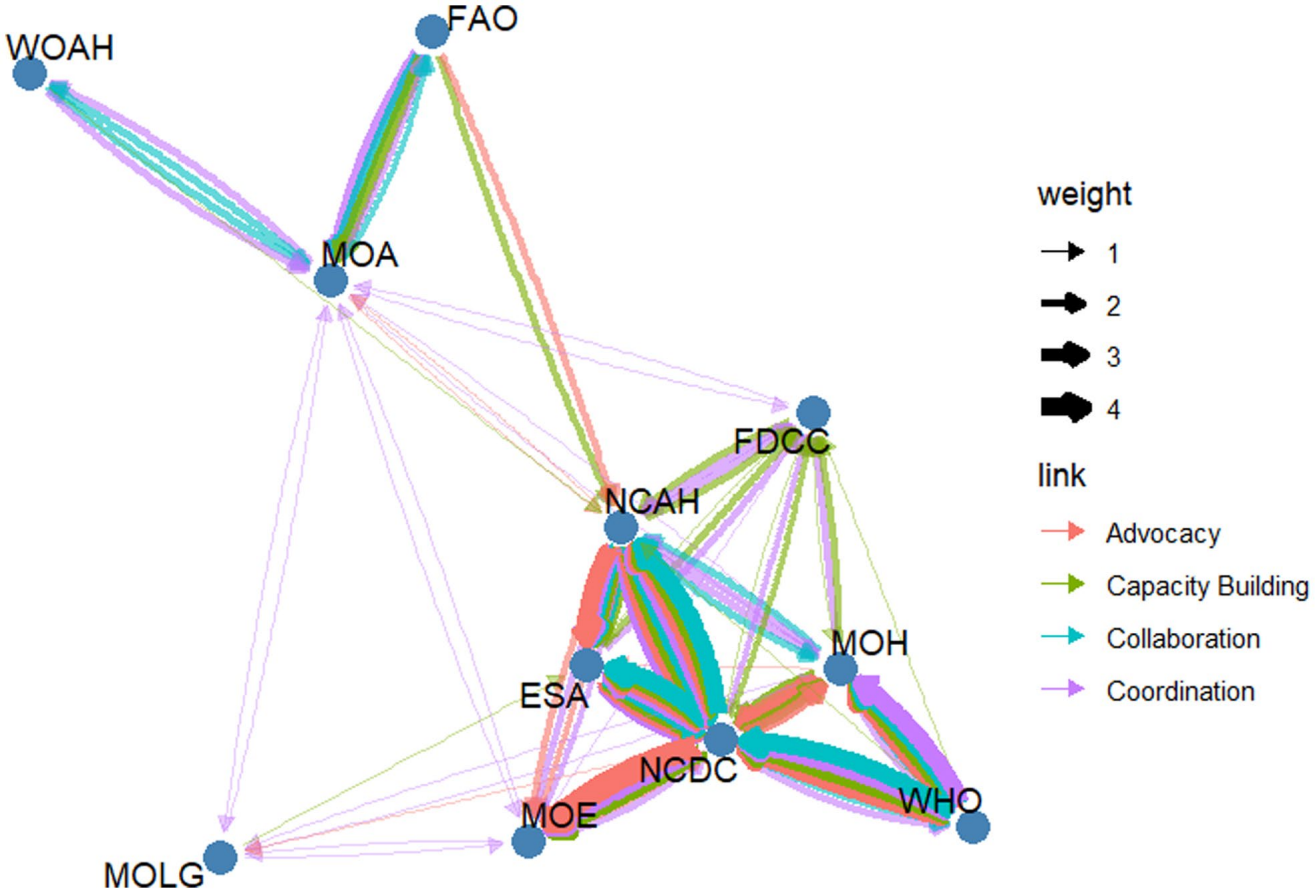
Network map of key stakeholders for One Health institutionalization in Libya.

Figure 2 presents the network map generated from the participatory Net-Map exercise, illustrating the structure and nature of relationships among key actors in Libya’s One Health network. The map identifies the NCDC and the National Center for Animal Health (NCAH) as the most connected nodes. The MoA was observed to be the primary connection point between international organizations (FAO, WOAH) and national agencies.

#### 3.2.1 Activity (engagement metrics)

Based on degree centrality, the NCDC, with 38 connections, and the NCAH, with 34 connections, are the core hubs of the network. The MoH, with 26 connections, also shows high centrality. Among the international organizations, the WHO emerges as the most connected actor, with 14 connections, approximately twice as many as WOAH and the FAO, with 5 and 7 connections respectively, both of which occupy more peripheral positions in the network. When considering weighted degree, the NCDC (128), NCAH (96), and ESA (68) emerge as the most intensively connected nodes in the network.

#### 3.2.2 Bridging (brokerage metrics)

Betweenness centrality highlights the MoA (0.334) as a critical bridge between network segments, followed by the FDCC (0.212) and the NCAH (0.152). In contrast, international organizations such as the WHO, the FAO, and the WOAH exhibit low betweenness centrality, indicating that they connect primarily to central hubs rather than serving as bridges between distinct clusters. The broker score explicitly quantifies the brokerage role suggested by betweenness centrality. The MoA had the highest broker score (0.223), confirming its critical function in liaising between the domestic operational cluster and the international agricultural governance cluster. The NCAH’s broker score (0.061) further supports its hybrid role as both a hub and an integrator between the human and animal health sectors. The analysis of constraint further clarifies the brokerage roles within the network. A high constraint score indicates that an organization’s partners are also highly interconnected with each other, limiting its room to maneuver or act as a broker. This is observed in entities like the ESA (0.795) and WOAH (0.823), whose influence is channeled through tight-knit groups. Conversely, the MoA exhibits a low constraint score (0.331), signifying its unique position in connecting otherwise disconnected groups and confirming its role as the network’s primary broker or “structural hole spanner.”

#### 3.2.3 Accessibility (Influence metrics)

In terms of closeness centrality, the MoA (0.667) and FDCC (0.625) demonstrate high accessibility. The WHO shows the highest accessibility (0.5) among the international organizations. These results indicate their ability to rapidly disseminate information or coordinate actions across the network. In contrast, international organizations such as FAO (0.333) and WOAH (0.357) exhibit low closeness centrality, reflecting their more peripheral positioning and limited direct reach to other stakeholders. Eigenvector centrality further underscores the central role of national technical institutions: the NCDC (1.000) and the NCAH (0.858) dominate the network, confirming that their influence stems not only from direct connections but also from their ties to other well-connected actors. The ESA (0.783) and the MoH (0.461) also hold notable influence within the core network. Conversely, WOAH (0.018) and FAO (0.055) have minimal eigenvector centrality, indicating limited integration into the network’s influential core and reinforcing their role as external supporters rather than central drivers of One Health coordination in Libya. Based on PageRank, the NCDC (0.210) and the NCAH (0.164) rank highest, reaffirming their roles as central, high-impact actors in Libya’s One Health ecosystem. In contrast, more peripheral entities—such as the MoLG (0.0281), FAO (0.0530), and WOAH (0.0357)—exhibit limited network prominence, reflecting their supportive rather than core coordinating functions (Table 1).

**Table 1:** Social network analysis metrics and derived roles for core One Health stakeholders in Libya.

Node	Degree of centrality	Weighted degree	Betweenness centrality	Broker Score	Constraint	Closeness	Eigenvector	Page rank	Role type
NCDC	38	128	0.005	0.003	0.462	0.435	1.000	0.210	Hub
NCAH	34	96	0.152	0.061	0.603	0.526	0.858	0.164	Hub-Broker
MoH	26	58	0.041	0.016	0.612	0.588	0.461	0.106	Hub-Broker
ESA	20	68	0.006	0.001	0.795	0.455	0.783	0.113	Hub
MoA	19	31	0.334	0.223	0.331	0.667	0.062	0.094	Hub-Broker
MoE	17	35	0.006	0.002	0.664	0.556	0.366	0.069	Peripheral
FDCC	16	24	0.212	0.078	0.633	0.625	0.215	0.051	Broker
WHO	14	42	0.013	0.003	0.762	0.500	0.393	0.076	Peripheral
MOLG	8	8	0.094	0.052	0.440	0.556	0.046	0.028	Broker
FAO	7	17	0.000	0.000	0.673	0.333	0.055	0.053	Peripheral
WOAH	5	9	0.000	0.000	0.823	0.357	0.018	0.036	Peripheral

NCDC, National Center for Disease Control; NCAH, National Center for Animal Health; MoH, Ministry of Health; ESA, Environmental Sanitation Affairs; MoA, Ministry of Agriculture; MoE, Ministry of Environment; FDCC, Food and Drug Control Center; WHO, World Health Organization; MOLG, Ministry of Local Government; FAO, Food and Agriculture Organization of the United Nations; WOAH, World Organization for Animal Health.

The network comprised 11 nodes (organizations) with a total of 102 edges (connections), representing multiple forms of interaction (coordination, collaboration, capacity building, and advocacy). The network exhibited a very short average path length of 2.05 and a diameter of 4, indicating that information can traverse the entire network efficiently. The transitivity score of 0.65 pointed to a high level of clustering, where organizations form tightly knit groups. The assortativity coefficient of −0.13 indicates a slight disassortative mixing pattern. This means that well-connected hubs (like NCDC and NCAH) tend to connect with less-connected, peripheral organizations. While this ‘hub-and-spoke’ structure enables efficient information flow through central actors, it also creates a potential vulnerability: the network’s resilience is highly dependent on its central hubs, making it susceptible to fragmentation if a key hub like the NCDC becomes incapacitated. The network is dominated by coordination links, which occur 40 times (39.2%) across 22 unique pairs. Capacity building is the second most frequent interaction, with 24 instances (23.5%) among 17 unique pairs. Advocacy appears 22 times (21.6%) with 12 unique pairs. Collaboration is the least common link type, recorded 16 times (15.7%) across 8 unique pairs Table 2.

**Table 2:** Global network metrics and distribution of interaction types in Libya’s One Health stakeholder network.

Category	Metric/link type	Value/count	Proportion	Description/unique pairs
Network Structure	Number of nodes (organizations)	11	-	The key stakeholder institutions in the network
	Number of edges (connections)	102	-	The total interactions between organizations
	Network diameter	4	-	The longest shortest path between any two organizations is 4 steps
	Average path length	2.05	-	On average, information travels between organizations in just over 2 steps
	Transitivity (clustering)	0.65	-	65% probability that two partners of an organization are also partners
	Assortativity	−0.13	-	Slight “hub-and-spoke” tendency in network structure
Link Types	Coordination	40	0.392	22 unique pairs
	Capacity building	24	0.235	17 unique pairs
	Advocacy	22	0.216	12 unique pairs
	Collaboration	16	0.157	8 unique pairs

### 3.3 Community structure and functional clusters

**Figure 3:**
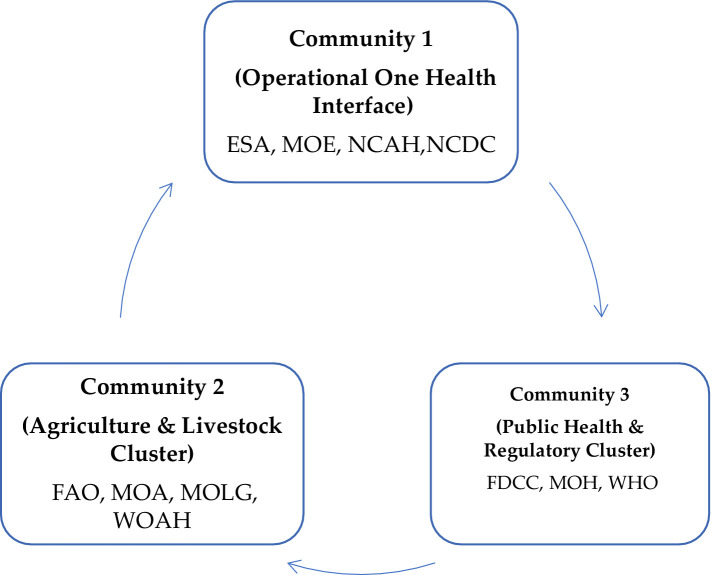
Community structure of Libya’s One Health stakeholder network revealed by the Louvain clustering algorithm. NCDC, National Center for Disease Control; NCAH, National Center for Animal Health; MoH, Ministry of Health; ESA, Environmental Sanitation Affairs; MoA, Ministry of Agriculture; MoE, Ministry of Environment; FDCC, Food and Drug Control Center; WHO, World Health Organization; MOLG, Ministry of Local Affairs; FAO, Food and Agriculture Organization of the United Nations; WOAH, World Organization for Animal Health.

Application of the Louvain algorithm to the stakeholder network identified three distinct communities with a modularity score of 0.195, indicating a statistically significant, non-random community structure Figure 3 (Supplementary Table 1)

- Community 1 includes the ESA, MoE, NCAH, and NCDC.
- Community 2 comprises the FAO, MoA, MoLG, and WOAH.
- Community 3 consists of the FDCC, MoH, and the WHO.

### 3.4 SWOT analysis findings

**Figure 4:**
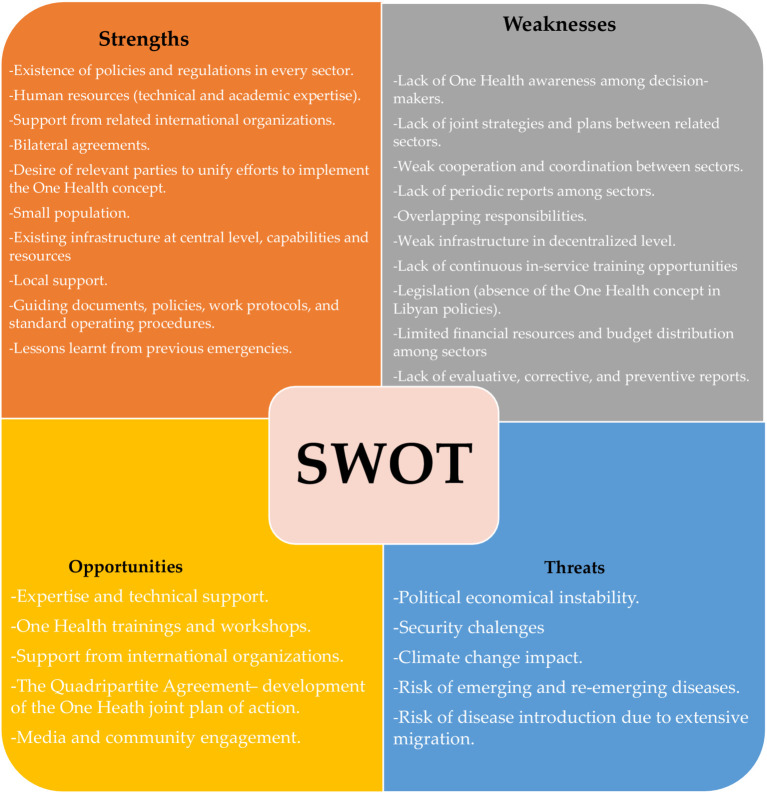
Strengths, weaknesses, opportunities, and threats (SWOT) analysis of factors influencing the implementation of the One Health.

The most significant strengths identified were the strong technical expertise of national institutions, the existence of centralized infrastructure, and a notable willingness among stakeholders to collaborate. Conversely, the most critical weaknesses included deeply fragmented intersectoral coordination, the absence of joint strategic plans, and limited financial resources dedicated to One Health activities. Key opportunities centered on the potential for alignment with the Quadripartite’s Joint Plan of Action and access to sustained international technical support. The most pressing threats were identified as the overarching political and economic instability, the impacts of climate change, and cross-border disease risks associated with migration Figure 4.

### 3.5 Development of the national one health memorandum of understanding (MoU)

The MoU was developed as a direct outcome of this stakeholder engagement process and established the formal governance framework for One Health in Libya. Its key provisions include:

- Signatory parties: The MoU is signed by the key “Manage Closely” institutions identified in the network analysis: the MoH, MoA, MoE, Food and FDCC, MoLG, and endorsed by NCDC and NCAH, in a ceremony facilitated by the WHO as a technical stakeholder.
- Governance structure: It mandates the establishment of a National One Health committee, with representation from all signatory parties. The High-Level Steering Committee is responsible for strategic oversight.
- Scope of collaboration: The MoU explicitly outlines priority areas for collaboration, which align with the workshop’s findings and the Quadripartite priorities. These include: (1) Joint epidemic surveillance and control of zoonotic diseases; (2) AMR containment; (3) Food safety; and (4) Addressing the human-animal-environment interface of climate change.
- Operational mechanisms: The framework commits parties to developing a joint plan and Standard Operating Procedures (SOPs) for outbreak preparedness and response, establishing a common data-sharing platform, and conducting regular joint simulation exercises.

## 4 Discussion

Since the early 2000s, when the concept of One Health was introduced, there has been a notable rise in initiatives to implement the approach globally, regionally, and nationally. Various entities such as governments, academia, and non-profit organizations have embraced the One Health philosophy, institutionalizing their commitment to cross-disciplinary and collaborative efforts via One Health frameworks, networks, steering committee, and technical groups, and task-forces (19).

This research provides the first in-depth stakeholder network analysis to guide the establishment of the One Health approach in Libya. Using participatory Net-Map exercises, SNA, and a SWOT evaluation, we mapped the governance landscape, identified systemic strengths and weaknesses, and contributed to developing a national One Health MoU. Our findings reveal a network marked by strong internal cohesion but limited cross-sector integration, with existing collaboration within domains such as human health, animal health, and agriculture, yet minimal intersectoral connectivity. This fragmentation aligns with a global review of One Health initiatives, which included 54 studies (77 programs). The study found that most initiatives involved only human and animal sectors, with little inclusion of the environmental sector. Nearly all programs emphasized policy and capacity building, while Pathway 2 (collaboration and engagement) was the most active, followed by Pathway 1 (policy, legislation, advocacy, and financing) and Pathway 3 (data, evidence, and knowledge). Both our study and the global review highlight a common gap in multisectoral integration, suggesting that despite active engagement and policy efforts, the One Health framework often remains fragmented across sectors (20). Furthermore, our findings on fragmentation and centralization find a revealing counterpoint in the experience of the Lao People’s Democratic Republic. While Libya’s network is structurally cohesive yet siloed, the network in Laos has been characterized as sparse and centralized, with core national organizations strained by numerous donor-driven projects (21). We suggest that two main factors lead to the structural differences observed. First, Libya’s strong pre-existing state capacity, bolstered by oil revenues, allows for a cohesive “hub-and-spoke” system. In contrast, Laos struggles with state capacity, resulting in a dispersed network reliant on international entities. Second, the nature of international intervention plays a role; Laos has many external donors, leading to competition and centralized administrative demands, while Libya benefits from a limited number of partners like the WHO and FAO, which support national centers through focused coordination. This observed fragmentation poses a significant risk that, in the face of zoonotic outbreaks or AMR threats—particularly in the post–COVID-19 context—responses may remain compartmentalized rather than fully coordinated (22, 23).

### 4.1 Central institutions and dual roles: strategic vs. operational leadership

The SNA findings indicate that the NCDC and the NCAH are fundamental to Libya’s One Health framework. Although both entities are central, their functions differ significantly: NCAH exhibits a balanced combination of influence and intermediacy (with high in-degree and betweenness), making it a key connector between human and animal health sectors. Conversely, NCDC stands out as the most active entity operationally (with the highest weighted out-degree), driving collaboration, capacity enhancement, and advocacy efforts. This contrast between strategic impact and operational engagement highlights the necessity for complementary leadership strategies in One Health governance. Among the international entities, the SNA results indicate that the WHO has the most operational entity (with the highest degree of centrality and weighted degree) in Libya.

The network displays a disassortative mixing pattern, with an assortativity of −0.13, leading to a ‘hub-and-spoke’ arrangement where central hubs primarily link to less-connected peripheral nodes. This structure provides efficiency and cohesion, which allow for effective coordination and rapid dissemination of information and resources from central nodes (such as NCDC and NCAH) to outer regions, enhancing leadership during routine operations. However, it introduces structural vulnerability; this efficiency renders the network at risk, especially if a central hub like the NCDC were to be removed. Such a loss could fragment the connections among peripheral nodes, causing disruption. This vulnerability is particularly alarming in Libya’s volatile context, where institutional stability is unpredictable.

Though the MoH does not get deeply involved in operations, it maintains a significant structural position due to its critical policy-making function. This observation is consistent with global patterns, where technical bodies typically lead execution and ministries offer strategic guidance (24).

Human-driven environmental changes—particularly agricultural intensification, deforestation, and ecosystem disruption—have led to increased encroachment into wildlife habitats, disrupting ecological balances and bringing humans and livestock into closer contact with wildlife reservoirs and disease vectors, thereby heightening the risk of infectious disease emergence and spread (25, 26). This highlights the essential role of MoA. In this research, MoA serves a crucial intermediary function, linking the domestic operational center (Community 1) with international standard-setting organizations (FAO, WOAH) in Community 2. Its significant betweenness centrality (0.334) and closeness (0.667) underscore its role as a channel for adapting global standards to national actions, a pattern also recognized in other LMICs where agriculture ministries lead zoonotic disease management (7).

#### 4.1.1 Community structure and functional clusters

The Louvain algorithm uncovered three functionally aligned groups within Libya’s health governance framework, revealing deeply ingrained institutional logics. Community 1, consisting of the NCDC, NCAH, ESA, and MoE, is characterized as an “Operational One Health Interface,” seamlessly integrating surveillance, field response, and environmental management at the junction of human, animal, and environmental health. This indicates that frontline integration is occurring naturally, without formal coordination mechanisms. Conversely, Community 2 acts as an “Agricultural and Livestock Governance Cluster,” with the MoA playing a pivotal role as a link between international standard-setting organizations (FAO, WOAH) and local policy (MoLG). Community 3 represents a “Public Health and Regulatory Cluster,” led by MoH, FDCC, and WHO, showcasing a robust command structure for human health regulation. Among the international bodies, the WHO acts as a major actor, indicating its pivotal role in not only coordinating but also pioneering the One Health approach in Libya. The WHO is mainstreaming the One Health approach across its technical units and country offices by providing strategic policy guidance, facilitating multisectoral coordination, and delivering targeted training at local, national, and regional levels—ultimately supporting country-led, sustainable One Health programming (27). However, the SNA shows the WHO’s influence as not widely visible from the perspective of the non-health sector, suggesting that its role is more catalytic than structurally central. Despite their internal cohesiveness, these clusters’ segmentation risks reinforcing isolated sectors. Thus, the national One Health MoU needs to extend beyond enhancing intra-cluster relations by intentionally creating mechanisms for cross-cluster collaboration. The MoA, with its notable betweenness centrality, serves as a strategic linchpin, bridging agricultural governance with public health and environmental operations. This data-informed community structure delivers a tailored blueprint for embedding One Health in Libya, suggesting that the governance framework can leverage existing collaborative networks while purposefully fostering connections among them, rather than applying a universal model.

### 4.2 Operationalizing cross-cluster collaboration: from structure to action

The identification of distinct clusters and key brokers provides a solid foundation for creating strategies to bridge sector gaps. To operationalize the strategic roles of these brokers, several mechanisms are recommended. First, the MoA should be utilized for policy bridging. With high betweenness centrality (0.334) but a low weighted degree (31), the MoA’s strength lies in connecting disparate parts of the network rather than in frequent interactions. Its role should be formalized as a policy facilitator, concentrating on developing integrated policies that align agricultural, public health, and environmental goals. Additionally, it should leverage its international ties (e.g., FAO, WOAH) to secure funding for cross-sector initiatives. Second, the NCAH needs to be empowered as an operational integrator. Given its high activity level (weighted degree = 96) and significant brokerage (betweenness = 0.152), the NCAH serves as an effective “hub-broker.” Its focus should be on creating standardized protocols for surveillance, laboratory testing, and data sharing, which will enhance collaboration between human and animal health sectors. Lastly, to mitigate structural risk, it is crucial to create redundancy within the network. The current disassortative structure (−0.13) and centralization around the NCDC and NCAH pose systemic risks. Therefore, the governance framework should promote direct connections by establishing multisectoral joint technical working groups (e.g., a “Zoonotic Disease Task Force”) and implementing a unified digital platform for disease surveillance. This strategy would aid in breaking down information silos and enhancing collaboration without the constant need for central intermediaries.

### 4.3 SWOT insights: building strengths, mitigating threats

The SWOT analysis contextualizes network findings within Libya’s operational reality. Key strengths—technical expertise, centralized infrastructure, and political will—provide a solid foundation. A global health risk framework is only as strong as the national public health infrastructure that forms its base, as these national systems are the first to confront pandemic threats and are therefore the essential foundation of our collective defense (28). However, key weaknesses, including fragmented coordination, the absence of joint intersectoral strategies, and limited capacity at decentralized levels, pose significant barriers to scaling up One Health implementation. Compounding these internal challenges are critical external threats such as political instability, climate change, and cross-border disease risks, all of which necessitate a resilient and adaptive governance framework. To effectively mitigate heightened risks and associated costs, policymakers must proactively address these vulnerabilities (29, 30). A successful adaptation strategy must be grounded in a robust conceptual understanding of the complex, multi-scale dynamics that shape health security in fragile contexts (31). The MoU’s focus on zoonotic surveillance, AMR containment, and climate-health interfaces directly responds to these systemic vulnerabilities.

### 4.4 Strengths, limitations, and future directions

This study represents the first comprehensive, mixed-methods stakeholder network analysis to inform One Health institutionalization in Libya. Its primary strength lies in the integration of participatory Net-Map exercises, quantitative SNA, and SWOT assessment—providing both qualitative depth and empirical rigor. The process directly engaged 42 participants from key national stakeholders across human, animal, and environmental health sectors, ensuring high contextual relevance and ownership. Critically, the findings were not merely diagnostic but were immediately operationalized into Libya’s national One Health MoU, demonstrating tangible policy impact. The use of multiple SNA metrics (degree, betweenness, eigenvector centrality, modularity, etc.) allowed for nuanced insights into both structural influence and operational engagement, revealing key brokers and functional clusters that would be invisible through simple stakeholder lists.

However, several limitations should be considered when interpreting these results, primarily stemming from the study’s scope and participant-defined boundaries. First, the purposive selection of national-level decision-makers and technical experts, while appropriate for mapping the core governance structure, led to the underrepresentation of subnational, private sector, and civil society actors. Consequently, the findings may not fully capture critical perspectives from frontline implementation, community engagement, and market-driven influences, potentially overrepresenting formal, government-led collaboration pathways. Second, the stakeholder network’s boundaries were defined by the workshop participants, resulting in the omission of influential international actors. Notably, entities with established environmental mandates, such as the United Nations Environment Program (UNEP) and the United Nations Development Program (UNDP), as well as key donors like the European Union and the Italian Agency for Development Cooperation, were not identified as central nodes. Their absence may obscure important sources of indirect influence, funding, and technical assistance that shape the network’s dynamics. As a result, the identified network structure represents a specific, top-down institutional perspective captured at a single point in time. This snapshot likely underestimates the complexity of the broader One Health landscape. For instance, including subnational actors might have revealed a more fragmented network, highlighting a policy-implementation disconnect. Similarly, the inclusion of UNEP and UNDP could have consolidated a stronger environmental cluster or identified a new broker for the climate-health nexus. Future research should deliberately incorporate these underrepresented groups to provide a more holistic, multi-level understanding of the One Health ecosystem in Libya and its capacity for decentralized execution and sustainable impact.

### 4.5 Policy implications and recommendations

To translate stakeholder network analysis into effective One Health governance in Libya, three key priorities are essential: First, institutionalizing national leadership by embedding the One Health within the governmental framework through a formal decree or mandate. This should include a dedicated budget and a transition plan to shift leadership from the WHO to a national agency, ensuring sustainability through domestic coordination. Second, bridging sectoral clusters by empowering the MoA and NCAH to connect under-engaged sectors, such as the MoE, particularly given the MoE’s role as the national lead for climate adaptation and resilience, as well as in the education and defense sectors. Developing formal inter-cluster protocols—like joint risk assessments and simulation exercises—is crucial to operationalize cross-sector collaboration. Third, establishing a results-oriented monitoring and evaluation (M&E) framework that tracks process and outcome indicators, such as joint planning meetings and budget allocations for One Health activities. This M&E system should be integrated into the national health information architecture and reported annually to enhance transparency and learning. These actions will strengthen Libya’s One Health system, making it resilient to political changes and health challenges.

## 5 Conclusion

The network is anchored by three pivotal institutions—the NCAH, NCDC, and MoH—which demonstrate complementary roles: NCAH as a strategic integrator, NCDC as an operational driver, and MoH as a policy leader. Critically, the MoA emerged as the key broker bridging domestic and international actors, a role now institutionalized through its co-leadership in the MoU. The network’s high reciprocity and short average path length reflect strong collaborative norms and efficient information flow within clusters. However, the tripartite community structure reveals a risk of sectoral fragmentation. The National One Health MoU directly addresses this by formalizing cross-cluster coordination mechanisms, joint surveillance, AMR containment, food safety protocols, and climate-health integration. Libya possesses significant strengths, including technical expertise, existing legislation, and centralized infrastructure. Its progress remains vulnerable to political instability, resource constraints, and weak decentralized capacity. Sustainable institutionalization will therefore require: (1) embedding the One Health within national governance with dedicated funding; (2) leveraging brokers like MoA and NCAH to connect under-engaged sectors (e.g., defense, finance, education); and (3) implementing a robust monitoring framework to track joint planning, multisectoral outbreak responses, and budget allocations. This network-informed approach offers a replicable model for One Health institutionalization in fragile and conflict-affected settings, demonstrating that even in contexts of instability, evidence-based stakeholder engagement can catalyze durable, multisectoral health governance.

## Data Availability

The original contributions presented in the study are included in the article/Supplementary material, further inquiries can be directed to the corresponding author.

## Glossary

AMR: Antimicrobial resistance
COHESA: Capacitating One Health in Eastern and Southern Africa
ESA: Environmental Sanitation Affairs
FAO: Food and Agriculture Organization of the United Nations
FDCC: Food and Drug Control Center
IFPRI: International Food Policy Research Institute
ILRI: International Livestock Research Institute
LMICs: Low- and Middle-Income Countries
MoA: Ministry of Agriculture
MoD: Ministry of Defense
MoE: Ministry of Environment
MoH: Ministry of Health
MoHE: Ministry of Higher Education
MoIA: Ministry of Interior Affairs
MoI: Ministry of Information
MoLG: Ministry of Local Government
MoSA: Ministry of Social Affairs
MoU: Memorandum of Understanding
NCAH: National Center for Animal Health
NCDC: National Center for Disease Control
NGOs: Non-governmental organizations
SNA: Social network analysis
SOPs: Standard operating procedures
SWOT: Strengths, Weaknesses, Opportunities, Threats
UNEP: United Nations Environment Program
UNDP: United Nations Development Program
WHO: World Health Organization
WOAH: World Organization for Animal Health
