# Supplementary material for: Toward One Health Institutionalization: harnessing stakeholder network as leverage to strengthen health security in Libya
Source: Front Public Health. 2025 Nov 21;13:1651901. doi: 10.3389/fpubh.2025.1651901 (PMC12678290; doi:10.3389/fpubh.2025.1651901)
Supplement: Supplementary file 1 [file Table_1.pdf]

Supplementary material:

Table 1: parameters of community cluster classification

Community	Size (No. of Nodes)	Average Degree*	Average Betweenness (Normalized)	Community Density†	Interpretation
Community 1 — Operational One Health Interface	4	27.2	0.0425	4.54	Highly connected cluster with strong operational coordination across sectors. High internal collaboration intensity, but relatively lower bridging role outside the cluster.
Community 2 — Agriculture & Livestock Cluster	4	9.75	0.107	1.62	Lower overall connectivity but the highest betweenness, indicating this cluster acts as a bridge between operational and regulatory sectors.
Community 3 — Public Health & Regulatory Cluster	3	18.7	0.0882	4.67	Moderately connected and internally cohesive cluster, suggesting effective coordination among policy and regulatory institutions.

* Average degree represents the mean number of weighted ties per node (multiple interaction types such as coordination, capacity building, and advocacy counted).
\† Density values represent **weighted connection strength**, not simple proportion of ties (thus may exceed 1.0). Higher density indicates stronger and more frequent internal collaboration.
